# NiO/TiO_2_ p-n Heterojunction Induced by Radiolysis for Photocatalytic Hydrogen Evolution

**DOI:** 10.3390/ma18153513

**Published:** 2025-07-26

**Authors:** Ana Andrea Méndez-Medrano, Xiaojiao Yuan, Diana Dragoe, Christophe Colbeau-Justin, José Luis Rodríguez López, Hynd Remita

**Affiliations:** 1 Institut de Chimie Physique, UMR 8000 CNRS, Université Paris-Saclay, 91405 Orsay, France; andy-mm@live.com (A.A.M.-M.); xyuan@iciq.es (X.Y.); christophe.colbeau-justin@universite-paris-saclay.fr (C.C.-J.); 2 Advanced Materials Department, Instituto Potosino de Investigación Científica y Tecnológica, A.C., IPICYT, San Luis Potosí 78216, SLP, Mexico; runlikehellpuma@gmail.com; 3 Institut de Chimie Moléculaire et des Matériaux d’Orsay, UMR 8182 CNRS, Université Paris-Saclay, 91405 Orsay, France; diana.dragoe@universite-paris-saclay.fr

**Keywords:** photocatalysts, hydrogen generation, solar fuels, nickel oxide nanoparticles, p-n heterojunction, radiolysis

## Abstract

Titanium dioxide (TiO_2_), a widely used semiconductor in photocatalysis owing to its adequate potential for water hydrolysis, chemical stability, low toxicity, and low cost. However, its efficiency is limited by fast charge-carrier recombination and poor visible light absorption. Coupling TiO_2_ with a *p*-type semiconductor, such as nickel oxide (NiO), forming a p-n heterojunction, decreases the recombination of charge carriers and increases photocatalytic activity. In this work, the surface of TiO_2_ modified with NiO nanoparticles (NPs) induced by radiolysis for photocatalytic hydrogen production was studied. The photocatalytic activity of NiO/TiO_2_ was evaluated using methanol as a hole scavenger under UV–visible light. All modified samples presented superior photocatalytic activity compared to bare TiO_2_. The dynamics of the charge carriers, a key electronic phenomenon in photocatalysis, was investigated by time-resolved microwave conductivity (TRMC). The results highlight the crucial role of Ni-based NPs modification in enhancing the separation of the charge carrier and activity under UV–visible irradiation. Furthermore, the results revealed that under visible irradiation, NiO-NPs inject electrons into the conduction band of titanium dioxide.

## 1. Introduction

The world urgently needs a green energy transition to combat rising greenhouse gases, which drive climate change and threaten ecosystems and human societies. The depletion of fossil fuel resources and their environmental impact underscore the need for sustainable energy alternatives. Furthermore, the shift to renewable energy sources like wind and solar is essential to mitigate climate change and ensure energy security. Thus, in this search for efficient and sustainable energy solutions, photocatalytic hydrogen production is a promising field that harnesses solar radiation, a renewable and intermittent energy source, to transform solar energy into chemical energy and store it in hydrogen bonds (H_2_). In this sense, semiconductor-based materials have garnered significant attention due to their potential applications in photocatalysis, photovoltaics, sensors, and ethanol oxidation technologies [1]. Among these materials, titanium dioxide (TiO_2_) and nickel oxide (NiO) stand out for their unique properties and versatility. TiO_2_ is well known for its adequate potential for water hydrolysis, low toxicity, low cost, and outstanding chemical and thermal stability [2], making it a prominent material for photocatalytic applications. However, its wide band gap (~3.2 eV for anatase and ~3.0 eV for rutile phases) [3] and fast charge-carriers’ recombination rate limit its efficiency under visible light. To overcome these limitations and improve the photocatalytic activity of TiO_2_, different strategies have been explored, including surface modification of titania with transition metals and plasmonic nanoparticles (NPs), transition metal oxides, graphitic carbon nitride, conjugated polymer nanostructures, etc. [4,5,6]. In particular, surface modification of TiO_2_ with noble metal NPs (e.g., Pt, Pd, Rh, Au, and Ag) can lead to important enhancement of the photocatalytic activity for H_2_ evolution [7,8,9,10,11,12]. Nevertheless, the elevated cost and limited availability of noble metals restrict their practical applications. Thus, there is a growing need to replace noble metals with abundant and cost-effective alternatives, such as transition metals. Earth-abundant metals, such as nickel (Ni)-based cocatalysts, are explored in photocatalysis because of their low cost and good efficiency [13,14]. Ni-based cocatalysts, such as metallic nickel (Ni^0^), nickel oxide (NiO) [12,15,16,17], and nickel hydroxide Ni(OH)_2_ nanoparticles [18,19], are very efficient cocatalysts when integrated with TiO_2_ semiconductor (SC) for hydrogen generation [20]. It has been observed that Ni^0^-NPs can be present at the TiO_2_/NiO-NPs interface and can lead to the formation of an ohmic junction with TiO_2_, which helps migration of photogenerated electrons to the metal [21,22]. NiO is a p-type SC with a band gap of about 3.5 eV [23,24,25,26], and forms, with TiO_2_, a p-n heterojunction that creates an internal electric field, reducing the charge-carriers’ recombination and facilitating interfacial charge transfer [21,23,27,28,29]. This p-n heterojunction plays a vital role in enhancing the photocatalytic activity of TiO_2_ under UV-vis light irradiation.

Accordingly, innovative materials such as NiO/TiO_2_ composites could play a crucial role in this transition to green energy by enhancing the efficiency and feasibility of renewable energy technologies. Recent studies have demonstrated that NiO/TiO_2_ composites exhibit superior performance in various applications, such as solar fuels’ generation and degradation of organic pollutants. Beyond these, NiO/TiO_2_ composites are increasingly being explored for different applications in contemporary industries, such as power generation, by enhancing the efficiency of photovoltaic cells to improve solar energy conversion rates; energy storage by developing advanced batteries and supercapacitors with higher capacity, longer lifespan, and faster charge–discharge cycles; and environmental remediation by efficiently degrading harmful pollutants in air and water. Industries ranging from automotive to consumer electronics are applying these advanced materials to drive innovation and improve product performance. For example, in the transportation sector, the development of NiO/TiO_2_-based materials can lead to more efficient fuel cells and batteries, directly impacting the efficiency and sustainability of electric and hybrid vehicles. In energy storage, the integration of these materials into batteries and supercapacitors promises significant advances in storage capacity and energy density, crucial for both consumer electronics and grid-scale energy solutions. Likewise, sensors and electronics will benefit from the creation of more sensitive and accurate sensors for gas detection and other environmental challenges.

In this work, the surface of TiO_2_ (commercial Degussa-P25) has been modified with Ni-NPs induced by radiolytic reduction of two different salt precursors, nickel (II) acetylacetonate and nickel (II) formate. Radiolytic reduction leads to a very homogeneous deposition of Ni-NPs on TiO_2_, obtaining a better deposition of NPs with the nickel (II) acetylacetonate precursor. The choice of the metal salt precursor used for the surface modification of TiO_2_ is essential to design efficient photocatalysts; therefore, through a comprehensive analysis of the structural, optical, and electronic properties of the nanomaterial, we seek to elucidate the mechanisms underlying the improved performance of NiO/TiO_2_ composites. When combined with TiO_2_, NiO can significantly improve the overall efficiency of the composite by enhancing charge separation, reducing recombination rates, and extending light absorption to the visible region. These improvements arise from the synergistic interactions between NiO and TiO_2_, which facilitate better utilization of a wider solar spectrum range and improved electron–hole pair dynamics, resulting in a good photocatalytic activity for H_2_ evolution under UV–visible light.

## 2. Materials and Methods

### 2.1. Chemical Reagents

Commercial titania (TiO_2_-P25, 80% anatase, and 20% rutile, Evonik, Essen, Germany) was used as photocatalytic support. Nickel (II) acetylacetonate (Ni(C_5_H_7_O_2_)_2_, Sigma-Aldrich, St. Louis, MO, USA, ≥99%) and nickel (II) formate dihydrate (Ni(HCO_2_)_2_•2H_2_O, Alfa Aesar, Lancashire, UK) were used as metal precursors. Ethanol (C_2_H_5_OH, VMR Chemicals, Radnor, PA, USA, 99.94%), methanol (CH_3_OH, Sigma Aldrich, ≥99%), deionized water (Milli-Q with 18.2 MΩ, Molsheim, France), and nitrogen gas (N_2_, Air Liquide, Paris, France) were used in all experiments.

### 2.2. Synthesis Method

**Figure 1:**
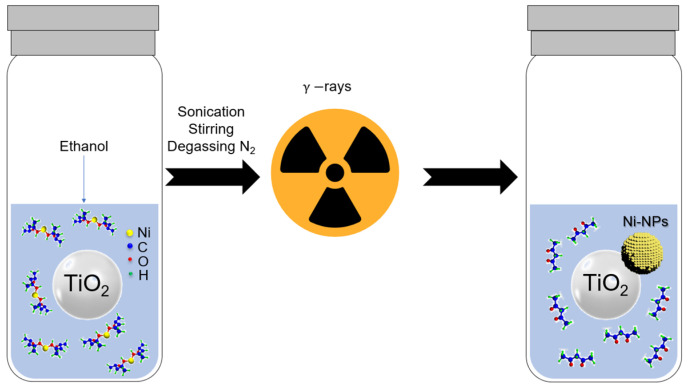
Synthesis of photocatalysts, Ni-based NPs/TiO_2_, by radiolysis method.

The photocatalysts were prepared by radiolysis (Figure 1), using two different metal precursors: nickel (II) acetylacetonate and nickel (II) formate. TiO_2_ was surface-modified with different amounts of Ni: the experimental design of metal loadings was 0.1, 0.5, 1.0, 3.5, and 5.0 wt.% Ni/TiO_2_. The required amounts of metal precursors were dissolved in ethanol to prepare each photocatalyst. The solutions were first sonicated for 10 min, stirred for 30 min, and degassed with N_2_ gas. The mixture was then irradiated using a ^60^Co panoramic gamma source. The doses used were sufficient for the complete reduction of Ni^II^ complexes to their zerovalent state Ni^0^ (Table S1). After the radiolysis process, the photocatalysts were separated by centrifugation. The powders were washed with ethanol 3 times at 6000 rpm for 10 min. The photocatalysts were then dried at 60 °C for 24 h. Radiolytic reduction is a powerful method to synthesize metal NPs in solutions and on supports using simple physicochemical conditions (ambient pressure and temperature, and no additional chemical-reducing agents) [30]. High-energy radiation (γ-rays, X-rays, electrons, or ion beams) of alcohols induces excitation and ionization of the solvent [31,32]. This process leads to the formation of solvated electrons ($e_{s}^{-}$) and alcohol radicals (${CH}_{3}C^{•}HOH$ in the case of ethanol) (see Equation (1)), which are strong reducing species. The reduction is very homogeneous in the medium with control of size and shape due to the high reducing power of solvated electrons and alcohol radicals [33]. Their redox potentials determined in water are $(E^{o}\left(\frac{H_{2}O}{e_{s}^{-}}\right))=-2.87V_{NHE}$ and $\left(E^{o}{(CH}_{3}CHO,\frac{H^{+}}{{CH}_{3}C^{•}HOH})\right)=-1.25V_{NHE}$, respectively [30,33]. This approach enables the reduction of metal complexes, which are challenging to reduce using conventional chemical methods at room temperature [31]. The solvated electrons and alcohol radicals reduce the Ni^II^ complexes to their zerovalent Ni^0^ state. The coalescence occurs, leading to the formation of metallic nanoparticles.

(1) $$C_{2}H_{5}OH⇝e_{s}^{-}+{CH}_{3}C^{•}HOH+C_{2}H_{5}OH_{2}^{+}$$

### 2.3. Characterization of NiO/TiO_2_

The morphology, size, and dispersion of nickel nanoparticles on the TiO_2_ surface were analyzed by transmission electron microscopy (TEM).

The crystal structure was analyzed by high-resolution transmission electron microscopy (HRTEM). Electron energy loss spectroscopy (EELS) was employed to determine elemental nickel and its oxidation states on the TiO_2_ surface using a FEI TECNAI F30 microscope (Hillsboro, OR, USA) equipped with a tungsten field emission gun operating at 300 keV.

Inductively coupled plasma optical emission spectrometry (ICP-OES) was used for elemental analysis, which provided information on the mass content of Ni on the TiO_2_ surface.

UV-vis diffuse reflectance spectroscopy (DRS) with a spectrophotometer (Cary 5000 Series, Agilent Technologies, Santa Clara, CA, USA) was used to investigate the optical properties of the samples. The measurements were conducted in the wavelength range of 200–800 nm.

The crystal structure of the synthesized photocatalysts was investigated by X-ray diffraction (XRD) using a SmartLab RIGAKU diffractometer (Tokyo, Japan) with Cu K_α_ radiation, 40 kV, 44 mA, λ = 0.15406 nm in the 2θ range from 20° to 80°, with steps of 0.01° s^−1^.

Furthermore, the surface elemental composition and oxidation states of nickel nanoparticles on the TiO_2_ surface were analyzed by X-ray photoelectron spectroscopy (XPS). XPS analysis was conducted using a ThermoFisher K-alpha spectrometer (Waltham, MA, USA) featuring a monochromated Al K_α_ X-ray Source (1486.68 eV) with a 400 µm spot size, providing an irradiated area of ~1 mm^2^. Measurements were carried out on powdered samples under a base pressure of 3 × 10^−9^ mbar. The hemispherical analyzer operated in constant analyzer energy mode with a pass energy of 200 eV and a step of 1 eV for the acquisition of survey spectra, and a pass energy of 50 eV and a step of 0.1 eV for the acquisition of narrow spectra. A “dual beam” flood gun was employed to neutralize the charge buildup. The binding energies were calibrated against the neutral carbon binding energy set at 284.8 eV. The precision in binding energy is ±0.2 eV. CasaXPS software (2.3.25 version) was used to record and treat the spectra [34]. The fitting procedure was carried out by first subtracting a Shirley-type background, followed by applying symmetrical line shapes for peaking fitting. The synthetic line shapes were sums of Gauss and Lorentzian functions with 30% Lorentzian character. Line shapes extracted from well-characterized Ni^0^, NiO, and Ni(OH)_2_ references were used for the fitting of Ni2p core-level spectra.

The time-resolved microwave conductivity (TRMC) technique was used to investigate the dynamics of charge carriers under UV and visible light excitations. The incident microwaves were generated by a Gunn diode (30 GHz). A tunable laser (EKSPLA, Vilnius, Lithuania, NT342B) between 200 and 2000 nm, equipped with an optical parametric oscillator (OPO), was used as a pulsed light source. The wavelengths used were 360 nm and 420 nm, with an excitation energy of 1.1 mJ and 2.3 mJ, respectively. When a semiconductor material is excited by a laser pulse, the TRMC technique is used to calculate the relative change ($\frac{∆P(t)}{p}$) in the microwave power reflected by the material. Equation (2) shows that this change ($∆\sigma \left(t\right)$) can be linked to a slight perturbation of the sample conductivity.

(2) $$\frac{∆P(t)}{p}=A∆\sigma \left(t\right)=Ae\mu_{e}∆n_{e}(t)$$

The primary data obtained from TRMC measurements include the maximum signal (*I_max_*), indicating the quantity of excess charge carriers induced by laser excitation, along with the subsequent decay, which reflects the reduction in free electron concentration over time The results obtained from this analysis reveal a reduction in the number of mobile electrons (indicated by a lower *I_max_* value) in the conduction band of the modified semiconductor, resulting in a shortened lifetime of the photogenerated electrons in the samples. Three phenomena related to metal deposition are responsible for this decrease in *I_max_*: (a) the shielding effect created by the NPs, (b) the surface recombination centers created by the synthesis process, and (c) the quick electron transfers from TiO_2_ to Ni-based NPs (occurring in less than 10 ns) [10,12]. These phenomena lead to the loss of charge carriers during the laser pulse. The principles of this technique and the phenomena have already been explained in previous articles [10,12,21,33,35,36,37].

### 2.4. Photocatalytic Hydrogen Generation Tests

The photocatalytic reactions were carried out in a sealed quartz reactor under vigorous stirring and an inert atmosphere. A total of 20 mg of the photocatalyst was dispersed in a 20 mL solution of 25% *v*/*v* methanol in water. Methanol was used as an electron donor to inhibit the oxidation reaction caused by the holes. Before irradiation, the reactor containing the photocatalyst in suspension (methanol/water) was degassed with nitrogen (N_2_) to remove oxygen (O_2_). Subsequently, the solution was exposed to UV–visible light using a Peschl photoreactor. The amount of H_2_ produced was measured by gas chromatography (Agilent System Technologies 7820A GC). A gas volume of 0.2 mL was injected into the gas chromatograph every hour for a duration of 5 h. For visible light experiments, a 300 W LOT-Oriel Xenon Lamp (250 to 2000 nm) equipped with a water filter (a large quartz cell) was positioned between the lamp and the reactor to block the infrared light and prevent sample heating. An optical filter was used to limit irradiation to the visible (λ ≥ 420 nm). The amount of hydrogen was measured by gas chromatography every hour for a duration of 5 h (Micro GC Fusion, INFICON, Bad Ragaz, Switzerland). The stability of the photocatalyst with cycling was also studied. After the photocatalytic test, the reactor was covered with aluminum foil and put in the dark. The next day, the reactor was degassed again and irradiated under the same conditions until 5 cycles were completed.

## 3. Results and Discussion

### 3.1. Characterization of the Photocatalysts

**Figure 2:**
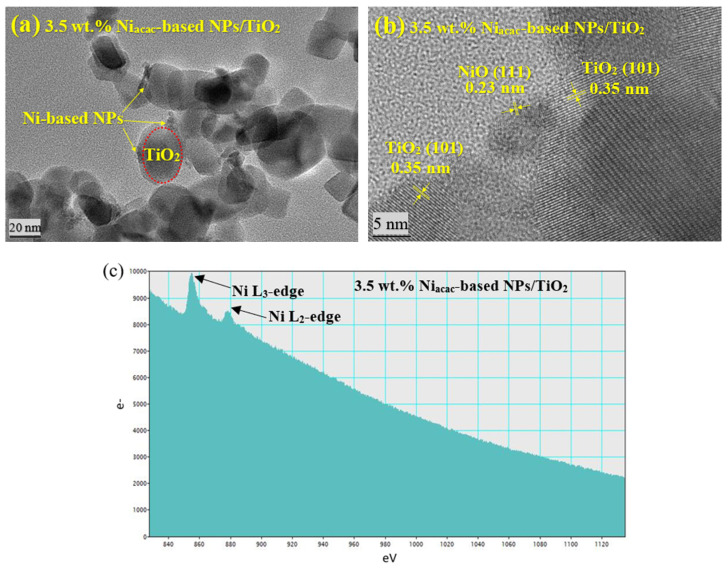
(**a**) TEM micrograph, (**b**) HRTEM micrograph, and (**c**) EELS spectrum of 3.5 wt.% Ni_acac_-based NPs/TiO_2_-modified sample.

The morphology, particle size, and distribution of Ni-NPs on the TiO_2_ surface were analyzed using TEM. Due to the similar atomic numbers of Ni and Ti, distinguishing the Ni nanoparticles by TEM was challenging. TEM images show that reduction of nickel (II) acetylacetonate precursor by radiolysis led to nanoparticles of about 8 nm for the 3.5 wt.% Ni_acac_-based NPs/TiO_2_ sample, as illustrated in Figure 2a. However, with Ni formate as the precursor, the Ni-based NPs are not distinguishable by TEM for the 3.5 wt.% Ni_formate_-based NPs/TiO_2_ sample (Figure S1a), which may be due to the small size of the NPs and low metal loading. The crystal structure was analyzed by HRTEM for the 3.5 wt.% Ni_acac_-based NPs/TiO_2_ sample (Figure 2b). The analysis revealed interplanar distances of 0.23 nm and 0.35 nm, corresponding to the lattice planes of NiO (111) and TiO_2_ anatase (101), respectively [21,38]. This result confirms the formation of a p-n heterojunction between NiO-NPs and titania. NiO-NPs are formed by back oxidation of Ni^0^-NPs (induced by radiolysis) when exposed to air during drying. Instead, the crystal structure of Ni-NPs was not detected in the 3.5 wt.% Ni_formate_-based NPs/TiO_2_ sample due to the small size of Ni-NPs (Figure S1b). Only an interplanar distance of 0.35 nm was observed, corresponding to the anatase phase of TiO_2_ (101).

EELS was used to reveal the presence of Ni and its oxidation states. Figure 2c displays the Ni-L_2_ and Ni-L_3_ edges at energy levels of 872 eV and 855 eV, respectively, for the 3.5 wt.% Ni_acac_-based NPs/TiO_2_ sample.

These edges are associated with the Ni^2+^ oxidation state [39]. The appearance of the L_2,3_ ionization edges of nickel is attributed to the excited internal electron transitions $p_{1/2}$ (L_2_ edge) and $p_{3/2}$ (L_3_ edge) to the empty valence states of character *s* and *d* [40,41]. In the case of the 3.5 wt.% Ni_formate_-based NPs/TiO_2_ sample (Figure S1c), no peak corresponding to the Ni-L_2_ and Ni-L_3_ edges was observed. ICP-OES was used for elemental analysis, providing insights into the mass content of Ni on the TiO_2_ surface. For 3.5 wt.% Ni_acac_-based NPs/TiO_2_ and 3.5 wt.% Ni_formate_-based NPs/TiO_2_ samples, the metal content is 1.30 wt% and 0.13 wt%, respectively. This result confirms that the precursor nickel (II) acetylacetonate is better than nickel (II) formate for the deposition of Ni-NPs on the TiO_2_ surface by radiolysis.

**Figure 3:**
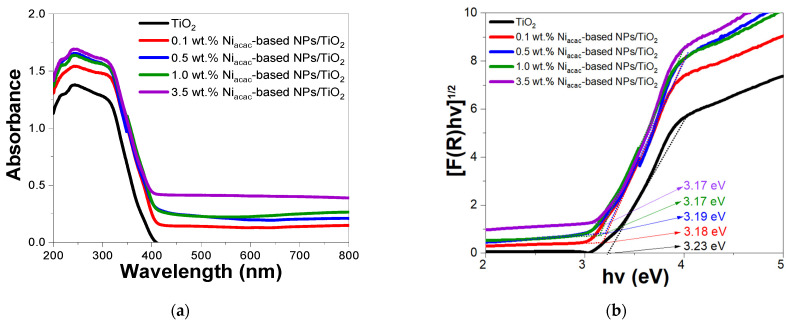
(**a**) DRS spectra and (**b**) their Tauc plot of Ni_acac_-based NPs/TiO_2_-modified samples and bare TiO_2_.

The optical properties of the modified photocatalysts and bare titanium dioxide were examined using UV-vis DRS. All samples showed strong absorption in the 200–400 nm range, which was attributed to the TiO_2_ support (Figure 3a and Figure S2a).

The modified samples showed a small redshift of the band edge compared to bare TiO_2_. The absorption in the visible range may be due to *d–d* transitions of Ni^2+^ [19,22,42,43,44] with Ni^0^-NPs being sensitive to oxygen. The band energy ($E_{g}$) can be calculated using the reflectance function F(R), where R is the reflectance; see Equation (3). The scattering and absorption coefficients are denoted by K and S, respectively. F(R) is proportional to the absorption coefficient (see Equation (4)), where h is Planck’s constant, ν is the photon frequency, $E_{g}$ is the energy of the band gap, and β is a factor depending on the transition probability and a constant in the optical frequency range. The factor *n* depends on the nature of the electron transition and takes a value of 2 or 1/2 for the direct and indirect transition band gaps, respectively [45].

(3) $$F\left(R\right)=\frac{{\left(1-R\right)}^{2}}{2R}=\frac{K}{S}$$

(4) $$(F(R)h\nu {)}^{n}=\beta \left(h\nu -E_{g}\right)$$

The band gap energies for the modified samples and bare TiO_2_ were calculated with the Tauc plot method, considering the indirect transition of anatase-phase TiO_2_ [3]. Figure 3b shows the calculated band gap energies for the bare TiO_2_ (3.23 eV), 0.1 (3.18 eV), 0.5 (3.19 eV), 1.0 (3.17 eV), and 3.5 (3.17 eV) wt.% Ni_acac_-based-NPs/TiO_2_ samples. Similarly, Figure S2b shows the band gap energies of the bare TiO_2_ (3.26 eV), 0.1 (3.26 eV), 0.5 (3.26 eV), 1.0 (3.26 eV), and 3.5 (3.18 eV) wt.% Ni_formate_-based NPs/TiO_2_ samples. A slight reduction in the band gap energy is observed for the Ni_acac_-modified samples, due to the higher content of Ni-based NPs deposited on the TiO_2_ surface, compared to the Ni_formate_-modified samples. From Figure 3a, it is worth noting that NiO-NPs exhibit absorption in the visible region [22,43,44].

The crystal structure of the photocatalysts was studied by XRD (Figure S3). The diffracted peaks of the samples coincide with the reference peaks of the anatase crystalline phase (JCPDS file No. 21-1272) peaks are found at 2*θ* values at 24.8°, 37.3°, 47.6°, 53.5°, 55.1°, and 62.2°; correspond to (101), (004), (200), (105), (211), and (204) planes. For the rutile crystalline phase (JCPDS file No. 21-1276), peaks are at 2*θ* values 27.0°, 35.6°, 40.8°, and 54.0°, corresponding to (110), (101), (200), and (211) planes [46].

No diffracted peaks corresponding to the crystalline phases of Ni-NPs were observed. This observation is consistent with previous studies, where researchers also reported the absence of nickel peaks due to the low adsorbed nickel content and very small size of Ni-NPs [19,28,42]. Furthermore, no shifts were observed in the diffraction peaks of the samples modified with Ni-NPs. This suggests that the presence of Ni-NPs has no effect on the lattice structure of TiO_2_, and that they are solely absorbed on the TiO_2_ surface.

**Figure 4:**
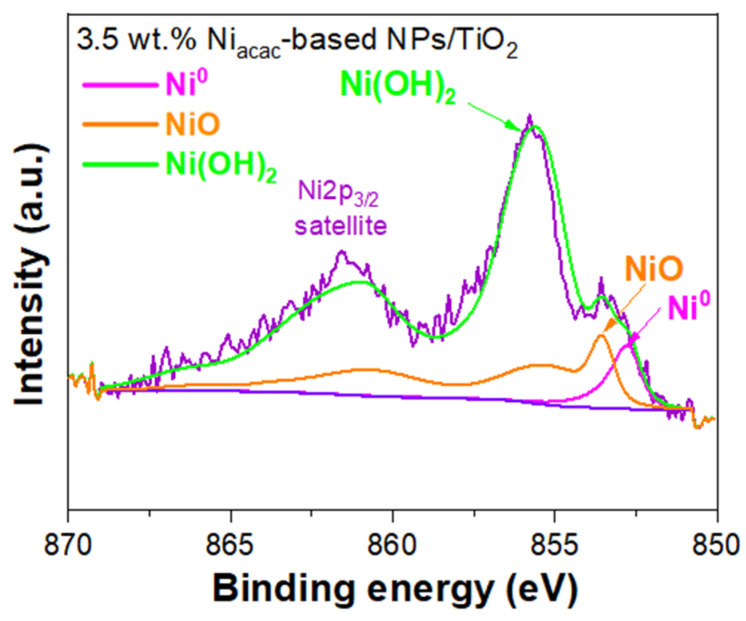
Narrow-scan XPS spectra of Ni *2p_3_*_/*2*_ of 3.5wt% Ni_acac_-based NPs/TiO_2_-modified sample.

XPS was used to examine the oxidation states and surface chemical composition of Ni-NPs on the TiO_2_ surface. The XPS survey for the 3.5 wt.% Ni_acac_-based NPs/TiO_2_ sample shows peaks corresponding to Ni, Ti, O, and C, indicating the presence of these elements on the surface of the modified sample; see Figure S4a. However, in the case of the 3.5 wt.% Ni_formate_-based NPs/TiO_2_ sample, only peaks for Ti, O, and C were detected (see Figure S4b). The absence of Ni peaks in the latter sample is due to the low content of Ni-NPs on the TiO_2_ surface, as determined by ICP-OES. The narrow-scan XPS spectra of the 3.5 wt.% Ni_acac_-based NPs/TiO_2_ sample were analyzed for Ni *2p_3_*_/*2*_, Ti *2p*, O *1s*, and C *1s* (Figure 4, Figure S5a, Figure S5b, and Figure S5c), respectively.

The Ni*2p_3_*_/*2*_ signal was fitted using synthetic line shapes and the fit parameters from the reference [47]. A Shirley-type background was subtracted from all spectra. The Ni*2p* signal shows contributions coming from Ni^0^ (852.7 eV), NiO (853.6 eV), and Ni(OH)_2_ (855.6 eV) on the TiO_2_ surface (Figure 4). The atomic percentages obtained for Ni^0^, NiO, and Ni(OH)_2_ are 6.63%, 33.60% and 59.77%, respectively. Ni (II) complexes have been reduced by radiolysis, leading to Ni^0^-NPs on TiO_2_. But these small NPs are sensitive to air and oxidize when exposed to air, which explains why the oxidized species are the main components of the Ni*2p_3_*_/*2*_ core-level spectrum. Ni^0^-NPs are expected to be located at the interface between NiO-NPs and TiO_2_, leading to the formation of an ohmic junction [21]. In the core level spectrum of Ti*2p* (Figure S5a), two symmetric peaks were observed at 458.7 eV and 464.5 eV, representing Ti *2p_3_*_/*2*_ and Ti *2p_1_*_/*2*_, respectively, which are the components of spin–orbit coupling [19,24]. The XPS spectrum of O *1s* (Figure S5b) displayed an asymmetric peak, likely arising from adsorbed hydroxide species [24,48]. The spectrum of O *1s* showed two peaks at 530.0 eV and 531.2 eV corresponding to lattice oxygen of TiO_2_ anatase and hydroxyl species, respectively [29,49]. The C *1s* spectrum (Figure S5c) showed the peaks at 284.8 eV, 286.0 eV, and 288.8 eV, corresponding to C-C, C-O, and C = O, respectively, attributed to carbon contamination [50].

**Figure 5:**
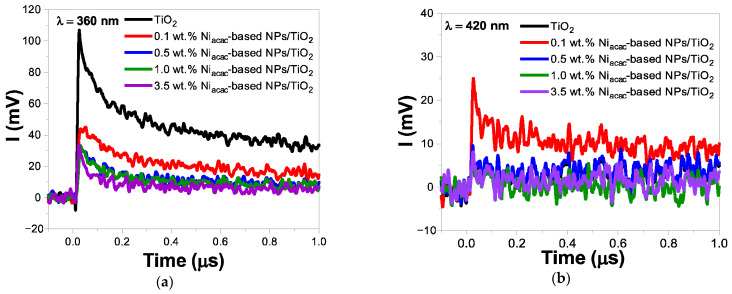
TRMC signals of Ni_acac_-based NPs/TiO_2_-modified samples and bare TiO_2_ at different wavelengths: (**a**) λ = 360 nm, and (**b**) λ = 420 nm. The laser energy of these wavelengths was 1.1 mJ and 2.3 mJ, respectively.

The TRMC signals highlight the significant impact of TiO_2_ surface modification with Ni-based NPs on the charge-carrier dynamics under UV and visible light excitation (Figure 5 and Figure S6). Just after the signal pulse, all samples reached *I_max_* values, indicating electron transfer from the valence band (VB) to the conduction band (CB) of TiO_2_. Simultaneously, NiO with a bandgap of 3.5 eV is also excited, and electron transfer from the VB of NiO to its CB occurs [21,22,51]. Subsequently, faster decays and reduced *I_max_* values were observed for the modified samples compared to bare TiO_2_ (Figure 5a and Figure S6a).

Electron transfer from titania to NiO-NPs is not thermodynamically allowed due to their higher position of CB level of NiO compared to the anatase phase of TiO_2_ [12]. However, the XPS results revealed a trace amount of Ni^0^ leading to the formation of an ohmic junction with TiO_2_. It is anticipated that these Ni^0^-NPs are located at the interface between NiO-NPs and TiO_2_, promoting the migration of photogenerated electrons to the metal [21,22]. The TRMC results provide strong evidence that Ni^0^-NPs effectively scavenge electrons from the conduction band of TiO_2_, which is beneficial for photocatalytic activity. [10,36,52] Simultaneously, the holes accumulated in the valence band of TiO_2_ will transfer to the NiO-VB, leading to more efficient charge-carrier separation [53]. In the case of the samples modified with nickel (II) acetylacetonate, the signal decay is faster than that of the nickel (II) formate salt precursor, and this signal decay is faster with increasing the metal loading (Figure 5a). The sample with 3.5 wt.% Ni_acac_-based NPs/TiO_2_ exhibits the fastest decay rate among the different metal loadings (0.1, 0.5, and 1 wt.%). In the case of formate precursor modified samples, only a small amount of Ni is deposited on the TiO_2_ surface. However, surface modification of TiO_2_ induces a decay of the TRMC signals (Figure S6a).

Furthermore, for the surface-modified TiO_2_ photocatalysts, detectable TRMC signals were observed under visible light excitation at 420 nm (Figure 5b and Figure S6b). This suggests that NiO-NPs are excited by visible light due to their *d–d* transitions of Ni^2+^ [19,42,43,44]. The transfer of the photogenerated electrons from the CB of NiO-NPs to the CB of TiO_2_ is facilitated. For Ni_acac_-modified samples, only 0.1 wt.% shows a detectable signal higher than that of bare TiO_2_ (Figure 5b). Nevertheless, for the 0.5, 1.0, and 3.5 wt.% samples, no detectable TRMC signals were obtained. This is likely due to the rapid electron transfer occurring within the system, and even if the TRMC technique allows us to obtain a nanosecond-scale signal, we cannot define the first 10 nanoseconds during the pulse. In the case of the Ni_formate_-modified samples, all the loadings show higher signals than bare TiO_2_ (Figure S6b).

### 3.2. Photocatalytic Hydrogen Generation

**Figure 6:**
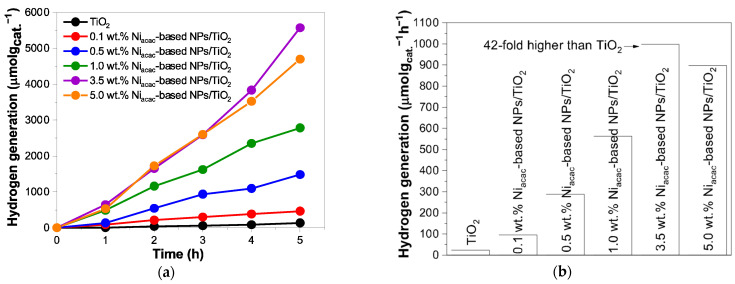
(**a**) Photocatalytic hydrogen generation for Ni_acac_-based NPs/TiO_2_ samples and bare TiO_2_ under UV–visible light, and (**b**) their hydrogen generation rates (µmol g_cat._^−1^ h^−1^) under UV–visible light irradiation from 25% *v*/*v* methanol aqueous solution.

The hydrogen generation is significantly enhanced when the TiO_2_ surface is modified with Ni-based NPs (obtained by radiolysis) under UV–visible light, as shown in Figure 6a.

Ni-based NPs act as active sites that promote hydrogen formation [12,20,21,22]. The p-n heterojunction formed between NiO-NPs and TiO_2_ induces an internal electric field that reduces charge-carrier recombination and facilitates interfacial charge transfer. The hydrogen evolution rate of TiO_2_ was 23.3 µmol g_cat._^−1^ h^−1^. The results demonstrate that the Ni_acac_-based NPs/TiO_2_ samples with theoretical Ni loadings of 0.1, 0.5, 1.0, 3.5, and 5.0 wt.% on the TiO_2_ surface exhibit increasingly higher and notable hydrogen generation rates, corresponding to 95.9, 287.8, 563.3, 998.4, and 897.2 µmol g_cat._^− 1^ h^−1^, respectively, as shown in Figure 6b. In particular, the most active sample was 3.5 wt.% Ni_acac_-based NPs/TiO_2_ (998.4 µmol g_cat._^−1^ h^−1^) after 5 h of irradiation, which is 42 times greater than that of bare TiO_2_ (23.3 µmol g_cat._^−1^ h^−1^). These outcomes highlight that the achievement of the highest H_2_ generation rates is dependent on optimal metal loading. This highlights the importance of carefully controlling the metal loading for optimal performance. The decrease in the activity with higher loading than 5.0% may be due to higher charge-carrier recombinations. However, the hydrogen generation rates with the Ni_formate_-based NPs/TiO_2_ samples were much lower compared to Ni_acac_-TiO_2_ because of the very small amount of Ni deposited with the Ni formate precursor, see Figure S7b. In fact, the deposition of Ni-based NPs on TiO_2_ is more effective with Ni acetylacetonate as precursor, as proved by the ICP-OES results. The samples 3.5 wt.% Ni_acac_-based NPs/TiO_2_ and 3.5 wt.% Ni_formate_-based NPs/TiO_2_ were tested under visible light, showing a slight activity with hydrogen generation rates of 1.5 and 0.6 µmol g_cat._^−1^ h^−1^, respectively. The sample 0.1 wt.% Ni_acac_-based NPs/TiO_2_ shows a higher TRMC signal than bare TiO_2_ (Figure 5b) under visible excitation, but the hydrogen generation rate (0.4 µmol g_cat._^−1^ h^−1^) was lower than that of 3.5 wt.% Ni_acac_-based NPs/TiO_2_. This may be due to a higher amount of NiO or NiOH and a lower amount of metallic Ni^0^ NPs, which may play the role of cocatalysts for H recombination and H_2_ formation.

**Figure 7:**
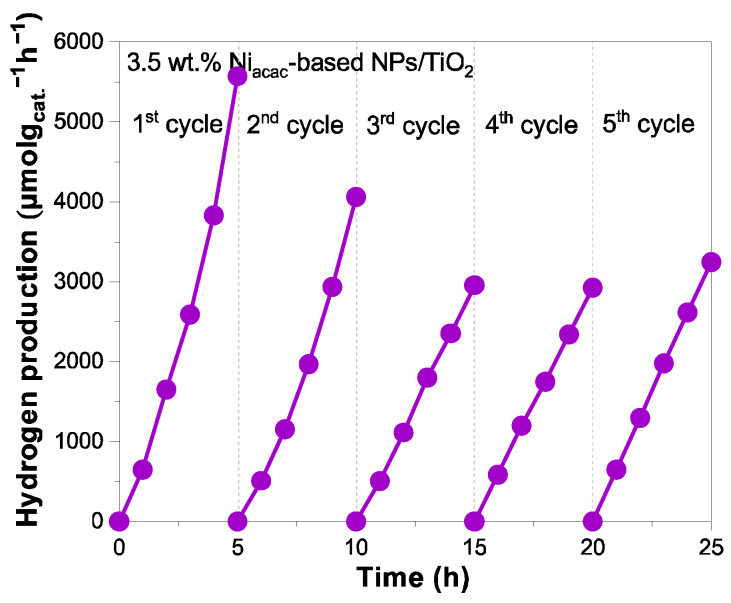
Photocatalyst stability with cycling for 3.5 wt.% Ni_acac_-based NPs/TiO_2_ sample under UV–visible light irradiation from 25% *v*/*v* methanol aqueous solution.

Finally, the photocatalytic stability with cycling of the best sample 3.5 wt.% Ni_acac_-based NPs/TiO_2_ is studied and presented in Figure 7.

**Figure 8:**
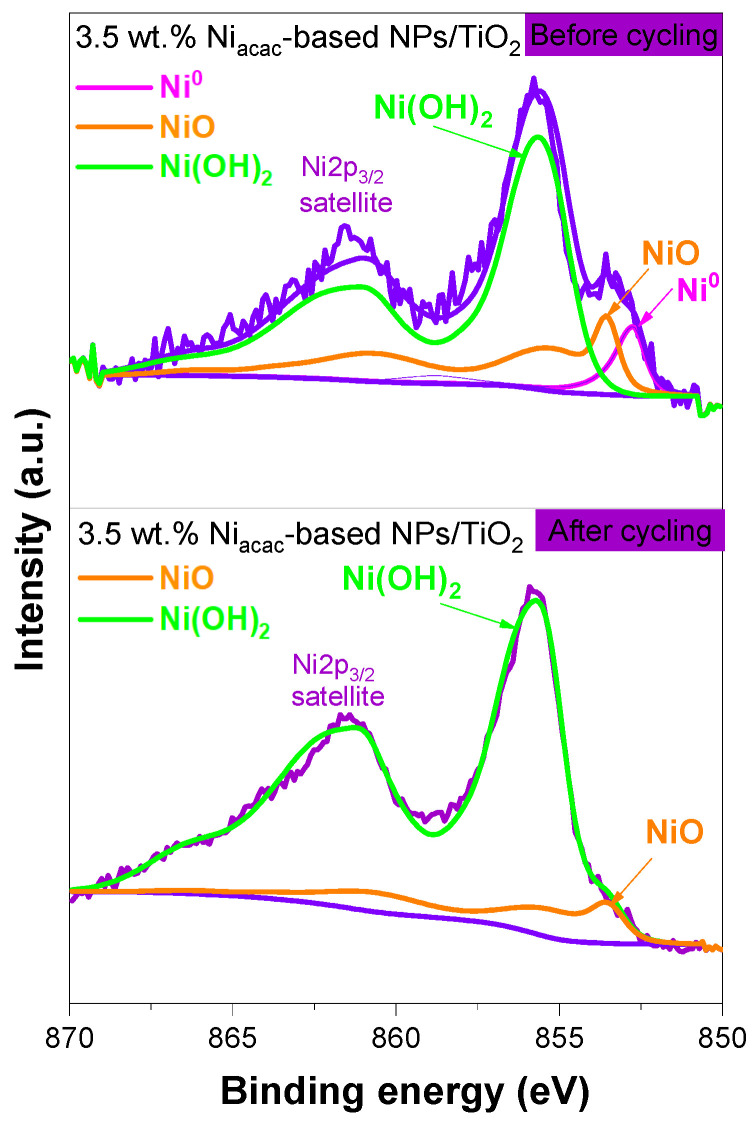
Narrow-scan XPS spectra of Ni2p*_3_*_/*2*_ of 3.5 wt.% Ni_acac_-based NPs/TiO_2_ sample before and after cycling.

The hydrogen generation decreases after the second cycle. This decrease may be due to nickel leaching or to the oxidation of the small Ni^0^ clusters. In fact, nickel leaching is known in the field of catalysis [38,54,55]. And one way to avoid this leaching in catalysis is to alloy Ni with noble metals [56,57]. After the third cycle, a plateau is obtained, evidencing photocatalytic stability, which is crucial for practical applications. The surface chemical composition and oxidation states of Ni-NPs on the TiO_2_ surface were analyzed by XPS before and after cycling. Figure S8 shows the XPS surveys of the 3.5 wt.% Ni_acac_-based NPs/TiO_2_ sample before and after five cycles. After cycling, the peak corresponding to Ni *2p* at 855.9 eV slightly decreases in intensity, confirming the nickel leaching. Figure 8 shows the comparison of the narrow-scan XPS spectra of Ni *2p_3_*_/*2*_ for the 3.5 wt.% Ni_acac_-based NPs/TiO_2_ sample before and after cycling. The Ni *2p_3_*_/*2*_ signal revealed the presence of NiO (853.6 eV) and Ni(OH)_2_ (855.7 eV) on the TiO_2_ surface. Ni^0^-NPs were not present after cycling.

Our results were compared with the literature on Ni-based NPs as cocatalysts on support semiconductors (Table S2). The comparison reveals notable differences in the efficiency of different catalysts under different conditions. The hydrogen generation rate of our best sample, 3.5 wt.% Ni_acac_-based NPs/TiO_2_, is 998.4 µmol g_cat._^−1^ h^−1^, ∼42-fold higher than that of bare TiO_2_ NPs, under UV–visible light irradiation. For comparison with other systems reported in the literature, Ni(OH)_2_/TiO_2_ nanotubes demonstrated approximately 12 times higher rate than TiO_2_ [19], and Ni/C/TiO_2_ showed 9 times higher rate than TiO_2_ [58]. NiO/CdS has been reported to be active under visible light (Table S2), with CdS being a semiconductor with a small band gap of 2.42 eV [59,60,61]. However, CdS is a toxic compound and tends to corrode under light irradiation, resulting in harmful Cd^2+^. For photocatalytic application, it is essential to use non-toxic and stable supports.

### 3.3. Proposed Photocatalytic Hydrogen Mechanism

**Figure 9:**
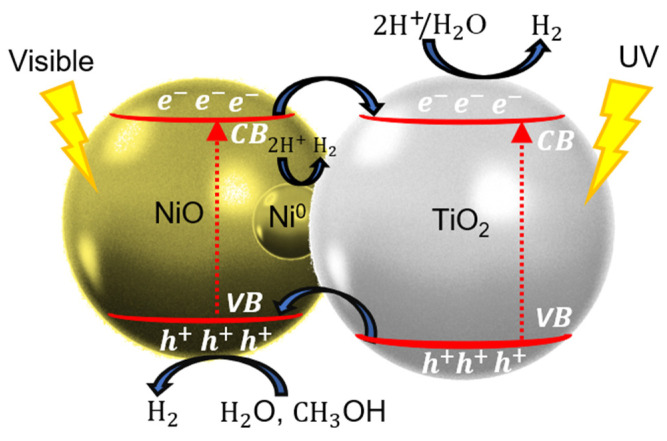
Photocatalytic mechanism of Ni-based NPs/TiO_2_ sample under UV–visible light excitation.

Hereby, we propose a photocatalytic hydrogen generation mechanism for the Ni-based NPs/TiO_2_ system under UV–visible light irradiation, as shown in Figure 9. However, the XPS analysis after cycling shows the disappearance of the Ni^0^ signal, with only NiO and Ni(OH)_2_ remaining detectable. This suggests that Ni^0^ is not stable under reaction conditions over multiple cycles, and this is associated with a decrease in H_2_ generation in the first three cycles. The mechanism probably shifts after the first couple of cycles, relying more heavily on the NiO-TiO_2_ p-n junction.

Recent research papers have investigated the photocatalytic hydrogen generation of p-n NiO/TiO_2_ heterojunction for H_2_ production using simulated solar light [16,17,21,25,62,63]. When NiO/TiO_2_ photocatalysts are exposed to solar light, electron–hole pairs are generated in both TiO_2_ and NiO semiconductors (Figure 9 and Equation (5)). In the photocatalytic process, the relative locations of the semiconductors’ CB and VB are crucial because they create an internal electric field that reduces charge-carriers’ recombinations and facilitates interfacial charge transfer. Electrons photogenerated in the NiO-CB migrate to the TiO_2_-CB, and holes photogenerated in TiO_2_-VB migrate to the NiO-VB (Equation (6)). This charge transfer decreases charge-carriers’ recombinations, leading to H_2_ production by proton (H^+^) reduction (Equation (7)) or water (H_2_O) reduction (Equation (8)). Photogenerated electrons reaching the surfaces of TiO_2_ and NiO-NPs can reduce H^+^ to H_2_.

Additionally, the TRMC results provide strong evidence that Ni^0^ NPs effectively scavenge electrons from the CB of TiO_2_ under UV light, which is beneficial for photocatalytic activity [36,52]. As we mentioned before, the XPS results revealed a trace amount of Ni^0^ leading to the formation of an ohmic junction with TiO_2_. These Ni^0^-NPs are located at the interface between NiO-NPs and TiO_2_, as shown in Figure 9, facilitating the migration of photogenerated electrons to the metal [21,22]. The photogenerated electrons reaching the surface Ni^0^-NPs can also reduce H^+^ to H_2_.

To prevent the oxidation reaction, a sacrificial agent is used: methanol (CH_3_OH). It effectively scavenges the holes of the VB of NiO to generate H_2_ [12,24]. Methanol and water molecules react with the holes, producing hydroxyl radicals (•OH) (see Equations (9) and (10)), which subsequently oxidize CH_3_OH molecules into formaldehyde (CH_2_O), formic acid (HCOOH), and carbon dioxide (CO_2_); see Equations (11)–(14). These compounds are the primary intermediates in the reforming of methanol to H_2_. The choice of methanol as a hole scavenger is advantageous owing to its high H/C ratio and the absence of C-C bonds, which reduces the risk of carbon formation and fouling of the photocatalyst [10,64]. Furthermore, the TRMC results under visible excitation confirmed detectable signals, indicating that NiO produces photogenerated electrons and holes due to its *d-d* transitions [19,22,42,43,44]. Under visible-light irradiation, these photogenerated electrons migrate from the CB of NiO to the CB of TiO_2_.

(5) $${TiO}_{2}+NiO\overset{hv>E_{g}}{\to }{TiO}_{2}\left(e_{CB}^{-}+h_{VB}^{+}\right)+NiO\left(e_{CB}^{-}+h_{VB}^{+}\right)$$

(6) $${TiO}_{2}\left(e_{CB}^{-}+h_{VB}^{+}\right)+NiO\left(e_{CB}^{-}+h_{VB}^{+}\right)\to {TiO}_{2}\left(e_{CB}^{-}\right)+NiO\left(h_{VB}^{+}\right)$$

(7) $${2H}^{+}+2e^{-}\to 2H^{•}\to H_{2}$$

(8) $$H_{2}O+e^{-}\to H_{2}+2{\mathrm{OH}}^{-}$$

(9) H2O+h+→OH•+H+

(10) CH3OH+h+→OH•+H+

(11) CH3OH+OH•→C•H2OH+H2O

(12) C•H2OH→CH2OH+H++e−

(13) $$CH_{2}O+H_{2}O\to HCOOH+H_{2}$$

(14) $$HCOOH\to {CO}_{2}+H_{2}$$

## 4. Conclusions

The modification of the TiO_2_ surface with Ni nanoparticles by radiolysis leads to small Ni-NPs dispersed on titania. The deposition of Ni-NPs with Ni acetylacetonate as a precursor is more efficient (compared to Ni formate), as confirmed by TEM, HRTEM, EELS, ICP-OES, DRS, and XPS techniques. The modified samples exhibited good photocatalytic activity for H_2_ evolution under UV–visible light and also slight activity under visible light. Notably, modification of TiO_2_ with the precursor nickel (II) acetylacetonate leads to higher hydrogen generation rates than that of nickel (II) formate. The Ni metal loading for Ni_acac_-modified samples was optimized for photocatalytic hydrogen generation performance. Among the modified samples, the 3.5 wt.% Ni_acac_-based NPs/TiO_2_ sample demonstrated the most outstanding increase in photocatalytic activity, with a rate approximately 42-fold higher than that of bare TiO_2_. The TRMC results provide strong evidence that Ni^0^-NPs effectively scavenge electrons from the CB of TiO_2_ under UV light, and Ni oxide NPs inject electrons into the conduction band of TiO_2_ under visible light. Additionally, the formation of p-n heterojunction between NiO-NPs and TiO_2_ played a crucial role in creating an internal electric field that reduced charge-carrier recombination and facilitated interfacial charge transfer. The modification of TiO_2_ with Ni-based NPs enhances charge-carrier separation and induces high H_2_ generation under UV–visible light. The photocatalytic activity reaches a stable rate after the second cycle. These findings advance our understanding of charge-carrier dynamics and provide valuable insights to optimize photocatalytic systems for sustainable energy applications. For further studies, we are developing photocatalysts with titania modified with bi- and tri-metallic alloys based on Ni. Alloying Ni with a noble metal such as Pt or Au is expected to enhance the photocatalytic activity and the stability of the photocatalyst.

## Data Availability

The original contributions presented in this study are included in the article/Supplementary Materials. Further inquiries can be directed to the corresponding author.

## Supplementary Materials

The following supporting information can be downloaded at https://www.mdpi.com/article/10.3390/ma18153513/s1, Figure S1: (a) TEM micrograph, (b) HRTEM micrograph, and (c) EELS spectrum of 3.5 wt.% Ni_formate_-based NPs/TiO_2_; Figure S2: (a) DRS spectra and their (b) Tauc plot of Ni_formate_-based NPs/TiO_2_-modified samples and bare TiO_2_; Figure S3: X-ray diffraction (XRD) patterns of (a) Ni_formate_-based NPs/TiO_2_ and (b) Ni_acac_-based NPs/TiO_2_-modified samples and bare TiO_2_ with the reference peaks of the anatase and rutile crystalline phases; Figure S4: Survey XPS spectra of (a) 3.5 wt% Ni_acac_-based NPs/TiO_2_ and (b) 3.5 wt% Ni_formate_-based NPs/TiO_2_ samples; Figure S5: Narrow-scan XPS spectra of (a) Ti *2p*, (b) O *1s*, and (c) C *1s* of 3.5 wt% Ni_acac_-based NPs/TiO_2_-modified sample; Figure S6: TRMC signals of Ni_formate_-based NPs/TiO_2_-modified samples and bare TiO_2_ at different wavelengths: (a) λ = 360 nm and (b) λ = 420 nm. The laser energy of these wavelengths was 1.1 and 2.3 mJ, respectively; Figure S7: (a) Photocatalytic hydrogen generation for Ni_formate_-based NPs/TiO_2_ samples and bare TiO_2_ under UV–visible light, and (b) their hydrogen generation rates (µmol g_cat._^−1^ h^−1^) under UV–visible light irradiation from 25% *v*/*v* methanol aqueous solution; Figure S8: Survey XPS spectra of 3.5 wt.% Ni_acac_-based NPs/TiO_2_ sample before and after cycling; Table S1: Doses for the modified samples; Table S2: Comparison of H_2_ evolution under UV–visible light of the different photocatalysts. References [15,19,23,24,28,42,43,58,59,60,61] are cited in the Supplementary Materials.

## Abbreviations

The following abbreviations are used in this manuscript:

H_2_	Hydrogen
TiO_2_	Titanium Dioxide
Ni	Nickel
NiO	Nickel Oxide
Ni^0^	Metallic Nickel
Ni(OH)_2_	Nickel Hydroxide
NPs	Nanoparticles
Acac	Acetylacetonate
N_2_	Nitrogen
O_2_	Oxygen
UV	Ultraviolet
CB	Conduction Band
VB	Valence Band
NHE	Normal Hydrogen Electrode
TEM	Transmission Electron Microscopy
HRTEM	High-Resolution Transmission Electron Microscopy
EELS	Electron Energy Loss Spectroscopy
ICP-OES	Inductively Coupled Plasma Optical Emission Spectrometry
DRS	Diffuse Reflectance Spectroscopy
XRD	X-Ray Diffraction
XPS	X-ray Photoelectron Spectroscopy
TRMC	Time-Resolved Microwave Conductivity
GC	*Gas Chromatography*
CONAHCYT	Consejo Nacional de Humanidades, Ciencias y Tecnologías
CNRS	Centre National de la Recherche Scientifique
UMR	Unité Mixte de Recherche
IPICYT	Instituto Potosino de Investigación Científica y Tecnológica
