# Supplementary material for: NiO/TiO2 p-n Heterojunction Induced by Radiolysis for Photocatalytic Hydrogen Evolution
Source: Materials (Basel). 2025 Jul 26;18(15):3513. doi: 10.3390/ma18153513 (PMC12348953; doi:10.3390/ma18153513)
Supplement: Supplementary file 1 [file materials-18-03513-s001.zip › materials-3702632-supplementary.pdf]

Supplementary Materials

Article

NiO/TiO2 p-n Heterojunction Induced by Radiolysis for Photocatalytic Hydrogen Evolution

Ana Andrea Méndez-Medrano [1,2], Xiaojiao Yuan [1,†], Diana Dragoe [3], Christophe Colbeau-Justin [1], José Luis Rodríguez López [2,‡] and Hynd Remita [1,*]

1 Institut de Chimie Physique, UMR 8000 CNRS, Université Paris-Saclay, 91405 Orsay, France; hynd.remita@universite-paris-saclay.fr
2 Advanced Materials Department, Instituto Potosino de Investigación Científica y Tecnológica, A.C., IPICYT, San Luis Potosí 78216, SLP, México; andy-mm@live.com
3 Institut de Chimie Moléculaire et des Matériaux d'Orsay, UMR 8182 CNRS, Université Paris-Saclay, 91405 Orsay, France; diana.dragoe@universite-paris-saclay.fr
* Correspondence: hynd.remita@universite-paris-saclay.fr
† Current address: Department of Chemistry, ICIQ, Institute of Chemical Research of Catalonia, 43007 Tarragona, Spain.
‡ Current address: Circ. Jardines de los Pinos, Jardines de Jacarandas, San Luis Potosí 78136, SLP, Mexico.

Table S1. Doses for the modified samples.

Sample	Concentration (M)	Dose (kGy)
0.1 wt.% Ni/TiO_2	5.5×10^{-5}	0.4
0.5 wt.% Ni/TiO_2	2.7×10^{-4}	1.8
1.0 wt.% Ni/TiO_2	4.5×10^{-4}	2.9
3.5 wt.%. Ni/TiO_2	1.8×10^{-3}	11.6
5.0 wt.%. Ni/TiO_2	2.2×10^{-3}	14.08

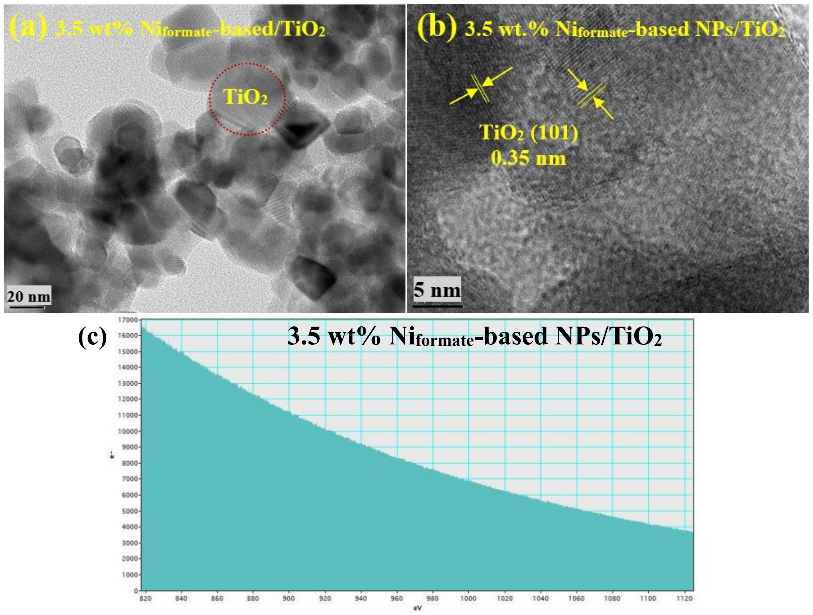

Figure S1. (a) TEM micrograph, (b) HRTEM micrograph, and (c) EELS spectrum of 3. 5 wt.% $Ni_{formate}$-based NPs/TiO_2.

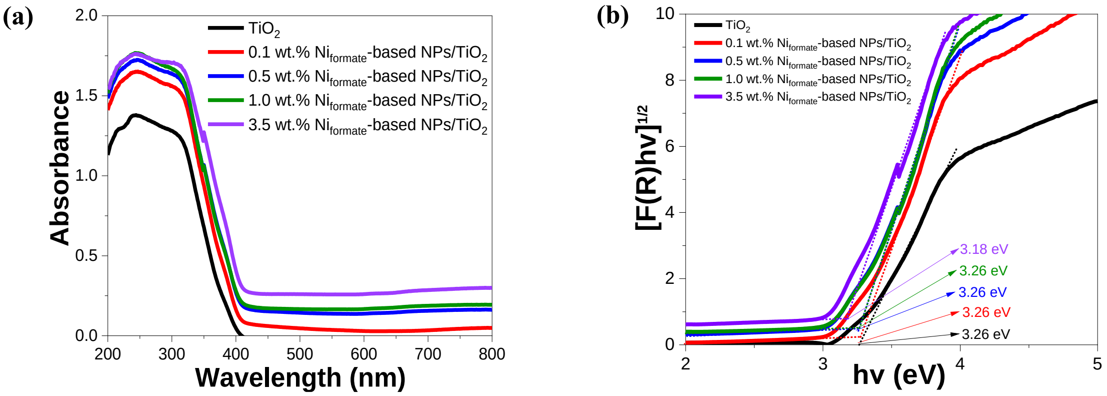

Figure S2. (a) DRS spectra and their (b) Tauc plot of $Ni_{formate}$-based NPs/TiO_2-modified samples and bare TiO_2.

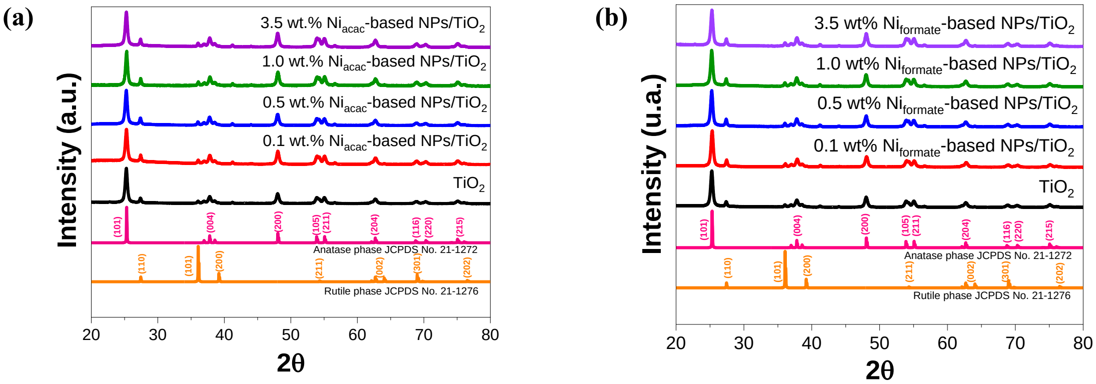

Figure S3. X-ray diffraction (XRD) patterns of (a) $Ni_{formate}$-based NPs/TiO_2 and (b) Ni_{acac}-based NPs/TiO_2-modified samples and bare TiO_2 with the reference peaks of the anatase and rutile crystalline phases.

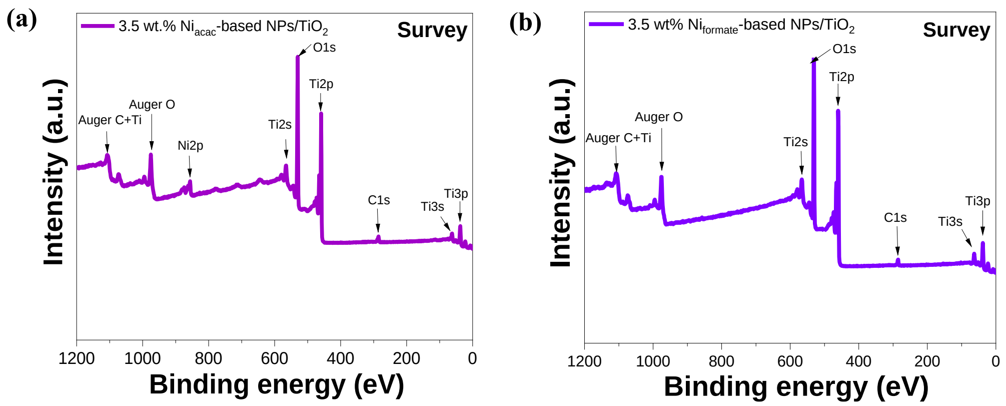

Figure S4. Survey XPS spectra of (a) 3.5wt% Ni$_{acac}$-based NPs/TiO_2 and (b) 3.5wt% Ni$_{formate}$-based NPs/TiO_2 samples.

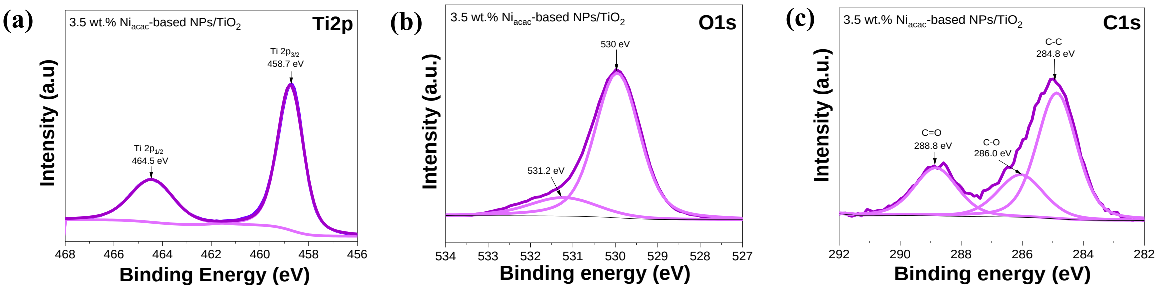

Figure S5. Narrow-scan XPS spectra of (a) Ti *2p*, (b) O *1s*, and (c) C *1s* of 3.5wt% Ni$_{acac}$-based NPs/TiO_2-modified sample.

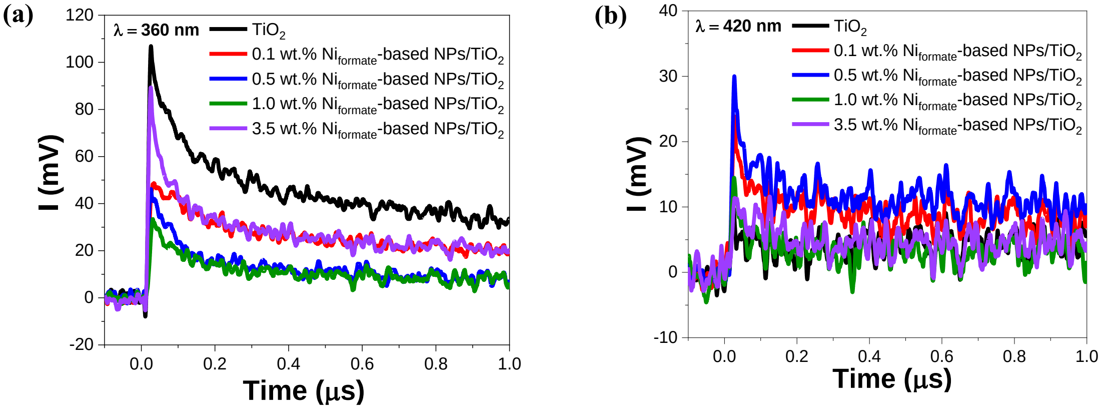

Figure S6. TRMC signals of Ni$_{formate}$-based NPs/TiO_2-modified samples and bare TiO_2 at different wavelengths: (a) λ=360 nm and (b) λ=420 nm. The laser energy of these wavelengths was 1.1 and 2.3 mJ, respectively.

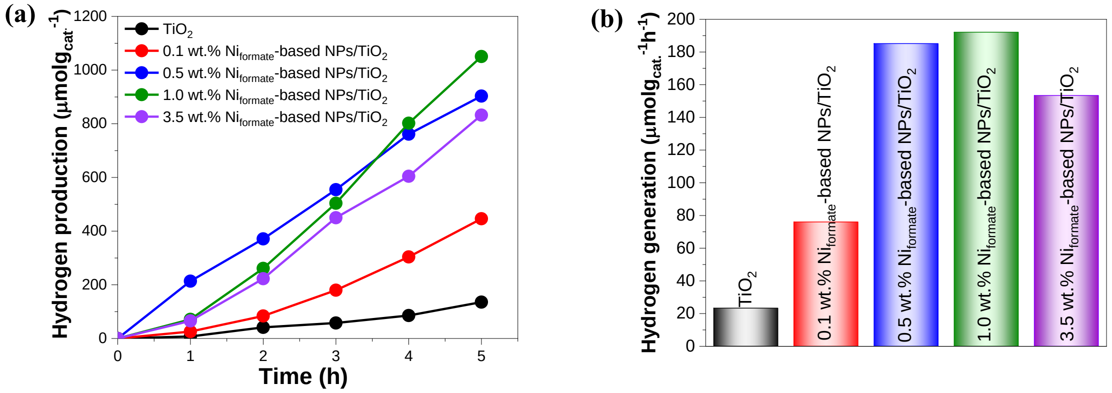

Figure S7. (a) Photocatalytic hydrogen generation for $Ni_{formate}$-based NPs/TiO_2 samples and bare TiO_2 under UV–visible light, and (b) their hydrogen generation rates ($\mu mol\ g_{cat.}^{-1}\ h^{-1}$) under UV–visible light irradiation from 25% v/v methanol aqueous solution.

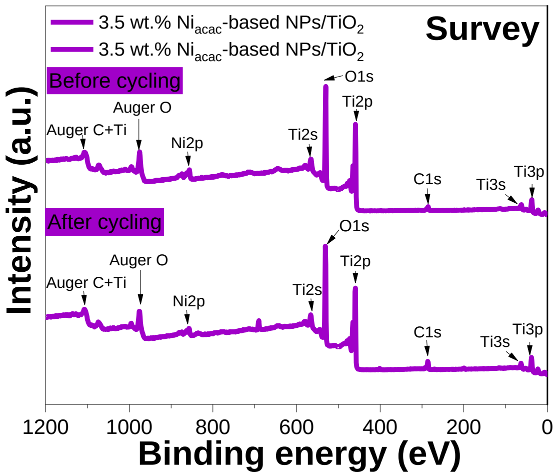

Figure S8. Survey XPS spectra of 3.5 wt.% Ni_{acac}-based NPs/TiO_2 sample before and after cycling.

Table S2. Comparison of hydrogen generation under UV–visible light of the different photocatalysts.

**Samples**	**Scavenger**	**Lamp**	**Hydrogen generation UV–visible**	**Hydrogen generation UV**	**Hydrogen generation Visible**	**Reference**
3.5 wt.% Ni-NPs/TiO_2	25% v/v methanol/water	Hg lamp	998.4 μmol $g_{cat.}^{-1}$ h^{-1} ~42-fold higher than TiO_2		1.5 μmol $g_{cat.}^{-1}$ h^{-1}	Our work
Ni/TiO_2	25% v/v methanol/water	Solarium Philips HB175 lamp Maximum peak at 365 nm		260.76 μmol h^{-1}		[1]
0.32 mol% Ni/TiO_2	25% v/v methanol/water	UV-LEDs at 365 nm	2547 μmol $g_{cat.}^{-1}$ h^{-1} ~135-fold than TiO_2			[2]
NiO/rGO/TiO_2	80 mL H_2O: 20 mL methanol	Solar simulator 300 W equipped with Xe lamp as source	240 μmol/h/g			[3]
2.0 wt % $Ni(OH)_2$/TiO_2 nanotubes	5% v/v crude glycerol/water	Solar light	4719 μmol$g_{cat.}^{-1}$$h^{-1}$ ~12-fold higher than TiO_2		0	[4]
Ni/C/TiO_2	80 mL H_2O: 20 mL methanol	300 W Xe lamp	1745 μmol$g_{cat.}^{-1}$$h^{-1}$ ~9-fold than TiO_2			[5]
1 wt% NiO/TiO_2	methanol/H_2O (1:1 v:v)	1. 6W Hg vapor light source wavelength range: 320-400 nm		2693 μmol $g_{cat.}^{-1}$ h^{-1}		[6]
0.5 wt% Ni/TiO_2-P25	Glycerol (10 vol%)	UV lamp at 365 nm		26000 μmol $g_{cat.}^{-1}$$h^{-1}$ >24 h		[7]
1.5 wt% NiO/TiO_2	200 mL water: 20 mLof methanol	300 W high-pressure Hg lamp		162. 6 μmol h^{-1}		[8]
NiO/CdS	100 mL of aqueous solution containing 30 mL methanol	Xe lamp (300 W) filter (λ > 400 nm)			445.6 μmol h^{-1} 41 times higher than CdS 5 h	[9]
Ni/GO-CdS	Ethanol:water (50/50 vol%)	4 LED lamps of λ=450 nm (4W each)			8866 μmol $g_{cat.}^{-1}$ h^{-1}	[10]
$Ni(OH)_2$-CdS/g-C_3N_4	20 mL aqueous solution containing Na_2S and Na_2SO_3	300 W Xe lamp 420 nm cut-off filter			115.18 μmol $mg_{cat.}^{-1}$ h^{-1}	[11]

References

1. Melián, E. P.; Suárez, M. N.; Jardiel, T.; Rodríguez, J. M. D.; Caballero, A. C.; Araña, J.; Calatayud, D. G.; Díaz, O. G. Influence of nickel in the hydrogen production activity of TiO_2. *Appl. Catal. B* **2014**, *152-153*, 192-201. DOI: 10.1016/j.apcatb.2014.01.039

2. Wang, W.; Liu, S.; Nie, L.; Cheng, B.; Yu, J. Enhanced photocatalytic H_2-production activity of TiO_2 using $Ni(NO_3)_2$ as an additive. *Phys. Chem. Chem. Phys.* **2013**, *15*, 12033–12039. DOI: 10.1039/c2cp43628k

3. Yu, X.; Zhang, J.; Zhao, Z.; Guo, W.; Qiu, J.; Mou, X.; Li, A.; Claverie, J. P.; Liu, H. NiO-TiO_2 p-n heterostructured nanocables bridged by zero-bandgap rGO for highly efficient photocatalytic water splitting. *Nano Energy* **2015**, *16*, 207-217. DOI: 10.1016/j.nanoen.2015.06.028

4. Lakshmana Reddy, N., Cheralathan, K. K., Durga Kumari, V., Neppolian, B., Muthukonda Venkatakrishnan, S. Photocatalytic Reforming of Biomass Derived Crude Glycerol in Water: A Sustainable Approach for Improved Hydrogen Generation Using $Ni(OH)_2$ Decorated TiO_2 Nanotubes under Solar Light Irradiation. *ACS Sustain. Chem. Eng.*, **6**, 3754, (2018). DOI: 10.1021/acssuschemeng.7b04118

5. Zhao, X.; Xie, W.; Shao, X.; Wang, Z.; Yang, B.; Yang, C.; Wang, J.; Su, X. An effective Ni/C co-catalyst for promoting photocatalytic hydrogen evolution over TiO_2 nanospheres. *Mater. Sci. Semicond. Process.* **2022**, *148*, 106775. DOI: 10.1016/j.mssp.2022.106775

6. Uddin, M. T.; Nicolas, Y.; Olivier, C.; Jaegermann, W.; Rockstroh, N.; Junge, H.; Toupance, T. Band alignment investigations of heterostructure NiO/TiO_2 nanomaterials used as efficient heterojunction earth-abundant metal oxide photocatalysts for hydrogen production. *Phys. Chem. Chem. Phys.* **2017**, *19*, 19279-19288. DOI: 10.1039/c7cp01300k

7. Chen, W. T.; Chan, A.; Sun-Waterhouse, D.; Llorca, J.; Idriss, H.; Waterhouse, G. I. N. Performance comparison of Ni/TiO_2 and Au/TiO_2 photocatalysts for H_2 production in different alcohol-water mixtures. *J. Catal.* **2018**, *367*, 27–42. DOI: 10.1016/j.jcat.2018.08.015

8. Sreethawong, T.; Suzuki, Y.; Yoshikawa, S. Photocatalytic evolution of hydrogen over mesoporous TiO_2 supported NiO photocatalyst prepared by single-step sol-gel process with surfactant template. *Int. J. Hydrog. Energy* **2005**, *30*, 1053-1062. DOI: 10.1016/j.ijhydene.2004.09.007

9. Chen, X.; Chen, W.; Lin, P.; Yang, Y.; Gao, H.; Yuan, J.; Shangguan, W. In situ photodeposition of nickel oxides on CdS for highly efficient hydrogen production via visible-light-driven photocatalysis. *Catal. Commun.* **2013**, *36*, 104–108. DOI: 10.1016/j.catcom.2013.03.016

10. Quiroz-Cardoso, O.; Oros-Ruiz, S.; Solís-Gómez, A.; López, R.; Gómez, R. Enhanced photocatalytic hydrogen production by CdS nanofibers modified with graphene oxide and nickel nanoparticles under visible light. *Fuel* **2019**, *237*, 227–235. DOI: 10.1016/j.fuel.2018.10.013

11. Yan, Z.; Sun, Z.; Liu, X.; Jia, H.; Du, P. Cadmium sulfide/graphitic carbon nitride heterostructure nanowire loading with a nickel hydroxide cocatalyst for highly efficient photocatalytic hydrogen production in water under visible light. *Nanoscale* **2016**, *8*, 4748–4756. DOI: 10.1039/c6nr00160b
